# Profiling abuse and neglect of women with disabilities: a step towards prevention of mistreatment of vulnerable populations

**DOI:** 10.3389/fgwh.2025.1580691

**Published:** 2025-09-01

**Authors:** Josephine Savard, Georgios Gavriilidis, Anna Lindblad, Jesse Huang, Milena Zeitelhofer Adzemovic

**Affiliations:** 1 Department of Clinical Neuroscience, Center for Molecular Medicine, Karolinska Institutet, Stockholm, Sweden; 2 Department of Clinical Science, Faculty of Medicine, Umeå University, Umeå, Sweden; 3 Centre for Healthcare Ethics, Department of Learning, Informatics, Management and Ethics, Karolinska Institutet, Stockholm, Sweden

**Keywords:** disability, violence, abuse, women's health, targeted interventions

## Abstract

Women with disabilities are at increased risk of violence and neglect, and the physical and psychological barriers to seeking help often lead to prolonged periods of abuse. In addition to being a leading cause of acute injuries and numerous chronic diseases, exposure to violence also negatively affects mental health. The aim of this cross-sectional quantitative data analysis was to investigate potentially distinct experiences of violence among women with disabilities resulting from cerebral palsy (CP), multiple sclerosis (MS), traumatic brain injury (TBI), stroke, arthritis as well as isolated sensory disabilities including visual- or hearing impairment. Indeed, our data shows that type of mistreatment, perpetrators and required personal assistance differ between disability groups. Interestingly, the highest frequency of violence/abuse was observed among women with hearing impairment. Together with MS, this type of disability was also more frequently associated with denial of help with basic needs or prevented use of assistive devices comparing to the other groups. Our results provide an insight into the types of abuse characteristic for certain disability groups, which can help develop more targeted preventive strategies. Furthermore, our findings indicate that prevalence of violence in certain disability groups remains unchanged despite societal efforts, hence calling for further research and more targeted interventions to prevent mistreatment of vulnerable populations.

## Introduction

The World Health Organization (WHO) has reported that approximately 30% of women in general population have experienced violence (1). Women with disabilities are subjected to prolonged periods of neglect and abuse, and at higher risk of victimization by multiple types of perpetrators (2, 3). Exposure to violence may also be associated with other risk factors, such as low socioeconomic status and education, which are not only related to unemployment and economic dependence but isolation and substance abuse (4–7). Moreover, violence is associated with many perpetrator-related characteristics, such as excessive alcohol consumption and patriarchal dominance, particularly jealousy and possessive behavior (4, 8).

Violence is consistently a leading cause of acute injury among women (5) and often correlates with worse long-term health outcomes, including increased risk of many chronic and stress-related diseases (9–11). It also negatively impacts mental health with higher rates of psychiatric disorders, including depression, generalized anxiety disorder, post-traumatic stress disorder, and drug and alcohol dependence (7, 12). In addition, disabilities pose physical and psychological barriers to seeking help, often leading to extended periods of abuse (11, 13). Despite an increased societal concern about abuse of women with disabilities, the extent of neglect and violence in this population has not been sufficiently investigated. This is reflected in unproportionally low number of studies and available databases that could be utilized in further, more specific investigations.

Expanding upon the findings of Milberger et al. (13), this study systematically examines the characteristics of abuse associated with physical disabilities across a range of medical conditions, including cerebral palsy (CP), multiple sclerosis (MS), traumatic brain injury (TBI), stroke, and arthritis, as well as hearing and visual impairments. Our aim was to further elucidate types of mistreatments in different disability types/diagnostic groups. Notably, our findings are not only in line with the latest reports, but they also provide further insight into the types of abuse associated with certain disability groups, which can help develop more specific strategies to address mistreatment in vulnerable populations.

## Method

### Cohort description

This study entails secondary analysis of data collected in 2000–2001 and published in 2003 by Milberger et al. with the aim to explore prevalence and risk factors for violence in adult women with physical disabilities (13). Mailings describing the study were sent to approximately 100 organizations primarily servicing those with physical disabilities, as well as to self-help groups, domestic abuse programs, social workers, and health care personnel across the state of Michigan, United States of America (USA). An informed consent was obtained from all subjects and/or their legal guardian(s). An initial interview was conducted either by phone or a self-administered questionnaire concerning any history of physical or sexual abuse, along with incidences where an individual prevented the use of an assistive device (e.g., wheelchair, cane, respirator) or refused to help with basic personal needs (e.g., medication, personal hygiene). To address risk factors for violence, women (>18 years old) who reported experiencing abuse were compared to those who did not report such history. Of the 177 enrolled participants, 100 women (56%) reported experiencing abuse, of which 85 women responded to a follow-up questionnaire detailing the history and relationships associated with their abuse. Data presented in the original study was analyzed both qualitatively and quantitatively (13).

**Figure 1:**
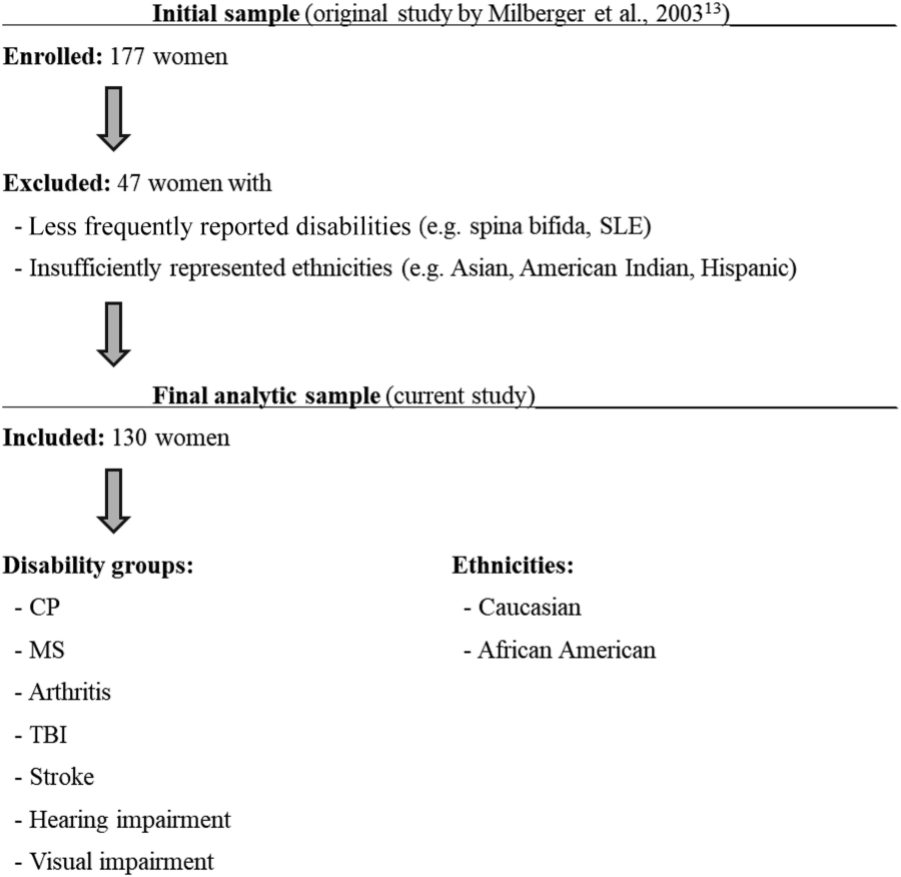
Flow chart illustrating participant enrollment to the original study (13) as well as inclusion and exclusion criteria applied to the original cohort for the purposes of the current study.

The present study extends towards evaluation of types of abuse among women with disabilities resulting from cerebral palsy (CP), multiple sclerosis (MS), arthritis, traumatic brain injury (TBI) and stroke. These groups were also compared against women with visual and hearing impairment. To enhance analytic clarity and statistical reliability, we applied specific inclusion and exclusion criteria to the original cohort (13). Of the 177 participants, individuals with rare disabilities such as spina bifida, systemic lupus erythematous (SLE), and post-polio syndrome were excluded due to insufficient representation. Additionally, participants who did not identify as either Caucasian or African American were excluded from the present analysis to avoid unstable subgroup estimates resulting from small sample sizes. Our final analytic sample consisted of 130 participants (see flow diagram in Figure 1). These categories included CP (*n* = 33), MS (*n* = 22), arthritis (*n* = 43), TBI (*n* = 13), stroke (*n* = 8), hearing impairment (*n* = 29), and visual impairment (*n* = 28). Of note, some individuals were qualifying under more than one diagnostic category/disability group. Demographic characteristics of the selected participants are summarized in Table 1.

**Table 1:** Descriptive statistics of cohort, stratified by disability-related diagnosis.

	Cerebral palsy *n* = 33	Multiple sclerosis *N* = 22	TBI/Stroke *n* = 19	Arthritis *n* = 43	Visual impairment *n* = 28	Hearing impairment *n* = 29
Age						
20–34 years	7 (21.2%)	0 (0.0%)	1 (5.3%)	3 (7.0%)	2 (7.1%)	7 (24.1%)
35–44 years	10 (30.3%)	13 (59.1%)	7 (36.8%)	9 (20.9%)	11 (39.3%)	7 (24.1%)
45–54 years	9 (27.3%)	8 (36.4%)	5 (26.3%)	14 (32.6%)	7 (25.0%)	7 (24.1%)
55+ years old	7 (21.2%)	1 (4.5%)	6 (31.6%)	17 (39.5%)	8 (28.6%)	8 (27.6%)
Ethnicity						
Caucasian	30 (90.9%)	16 (72.7%)	17 (89.5%)	37 (86.0%)	21 (75.0%)	26 (89.7%)
African Ame	3 (9.1%)	6 (27.3%)	2 (10.5%)	6 (14.0%)	7 (25.0%)	3 (10.3%)
Living condition						
Independent	23 (71.9%)	19 (86.4%)	10 (55.6%)	33 (76.7%)	23 (82.1%)	21 (72.4%)
Semi-indep.	8 (25.0%)	3 (13.6%)	3 (16.7%)	8 (18.6%)	2 (7.1%)	4 (13.8%)
Other	1 (3.1%)	0 (0.0%)	5 (27.8%)	2 (4.7%)	3 (10.7%)	4 (13.8%)
Marital status						
Married	5 (15.2%)	8 (36.4%)	3 (15.8%)	6 (14.3%)	9 (32.1%)	7 (24.1%)
Single	20 (60.6%)	3 (13.6%)	8 (42.1%)	18 (42.9%)	11 (39.3%)	14 (48.3%)
Divorced	6 (18.2%)	9 (40.9%)	5 (26.3%)	13 (31.0%)	7 (25.0%)	6 (20.7%)
Widowed	2 (6.1%)	2 (9.1%)	3 (15.8%)	5 (11.9%)	1 (3.6%)	2 (6.9%)
Employment						
Full-time	4 (12.1%)	2 (9.1%)	0 (0.0%)	6 (14.0%)	6 (21.4%)	5 (17.2%)
Part-time	8 (24.2%)	2 (9.1%)	5 (26.3%)	7 (16.3%)	9 (32.1%)	5 (17.2%)
Retired	4 (12.1%)	3 (13.6%)	3 (15.8%)	6 (14.0%)	4 (14.3%)	4 (13.8%)
Unemployed	13 (39.4%)	13 (59.1%)	9 (47.4%)	16 (37.2%)	5 (17.9%)	12 (41.4%)
Other	4 (12.1%)	2 (9.1%)	2 (10.5%)	8 (18.6%)	4 (14.3%)	3 (10.3%)

* Traumatic brain injury (TBI).

### Data analyses

Differences in the frequency of measure for abuse and physical impairment between groups were assessed using Fischer's exact test. All analyses and illustrations were performed with R (v.4.2.1).

### Ethical statement

The study protocols were approved by the Investigative Ethical Review Board at Wayne State University and was carried out in accordance with relevant national and state guidelines and regulations. All study participants have provided informed and written consent prior to enrollment. The current study utilized a dataset that is freely available online through the Michigan government's public repository: https://doi.org/10.3886/ICPSR03414.v1

## Results

**Figure 2:**
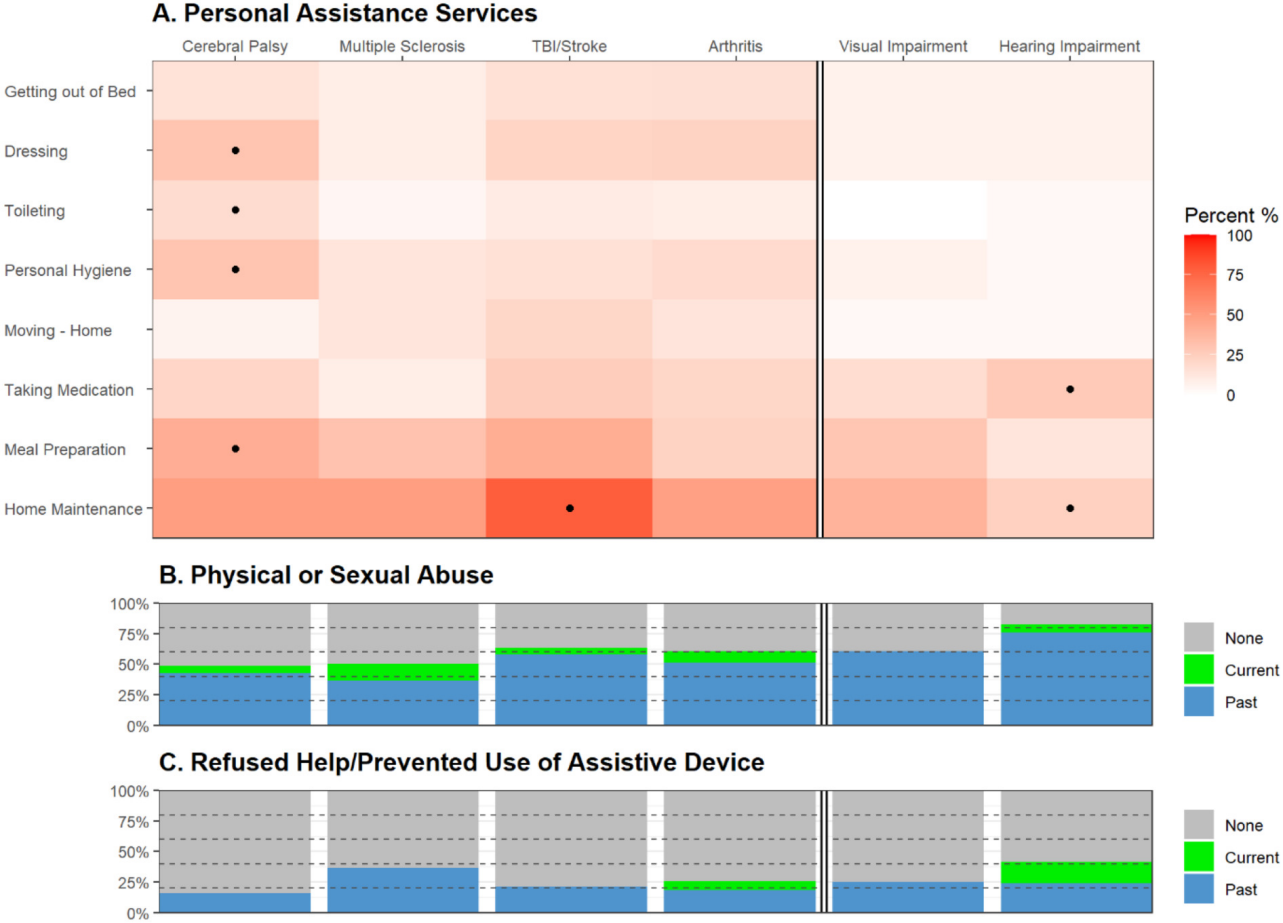
Physical and sexual abuse among women with disabilities. Heatmap **(A)** illustrates frequency rate (%) of self-reported use of personal assistance services. Frequencies were compared between disability groups using Fisher's exact test with *P* < 0.05 highlighted (dot). Stacked barplots illustrate the frequency rate (%) of **(B)** physical or sexual abuse and **(C)** incidences when they were refused help or was prevented from the use of an assistive device (current, past, or none). TBI, traumatic brain injury.

Most participants were between the ages of 35–54 at the time of enrollment; only those with arthritis were generally older than in the other groups (45+ years old). Most women were unemployed or retired (53.8%), with the highest unemployment rate among women with MS (59.1%). Many also lived independently (78.1%), single or divorced at enrollment, with more than half requiring personal assistance (56.9%, Figure 2A). Women with CP were more likely to require assistance services for dressing (30.3%, *p* = 0.01), toileting (18.2%, *p* = 0.02), personal hygiene (30.3%, *p* = 0.008), and meal preparation (42.4%, *p* = 0.01). Assistance with home maintenance was more common among women with TBI and stroke (78.9%, *p* = 0.0009), while those with hearing impairment were more likely to require assistance with taking medication (27.6%, *p* = 0.04).

Most women (57.7%) had experienced physical or sexual abuse as adults, with 7.7% reporting ongoing abuse at the time of response (Figure 2B). Interestingly, the highest frequency of abuse was observed among those with hearing impairment (82.8%, *p* = 0.002), followed by TBI and stroke (63.2%), visual impairment (60.7%), arthritis (60.5%), MS (50.0%), and lastly CP (48.5%). Approximately a quarter of the study participants also reported being denied help with basic needs or prevented from using assistive devices (Figure 2C). This was more commonly associated with hearing impairment (41.4%, *p* = 0.03) and MS (36.3%). Notably, more women with hearing impairment reported ongoing incidences at the time of response (17.2%, *p* = 0.006). Although the causes of visual and hearing impairment were unspecified, women with hearing impairments had a slightly higher proportion of arthritis, indicating age may play a role in some cases. Similarly, women with visual impairments had a greater frequency of neurological conditions, including MS (13.3%), TBI (13.3%), and spinal cord injury (6.7%). However, these trends were merely suggestive of the baseline characteristics of the study participants.

**Figure 3:**
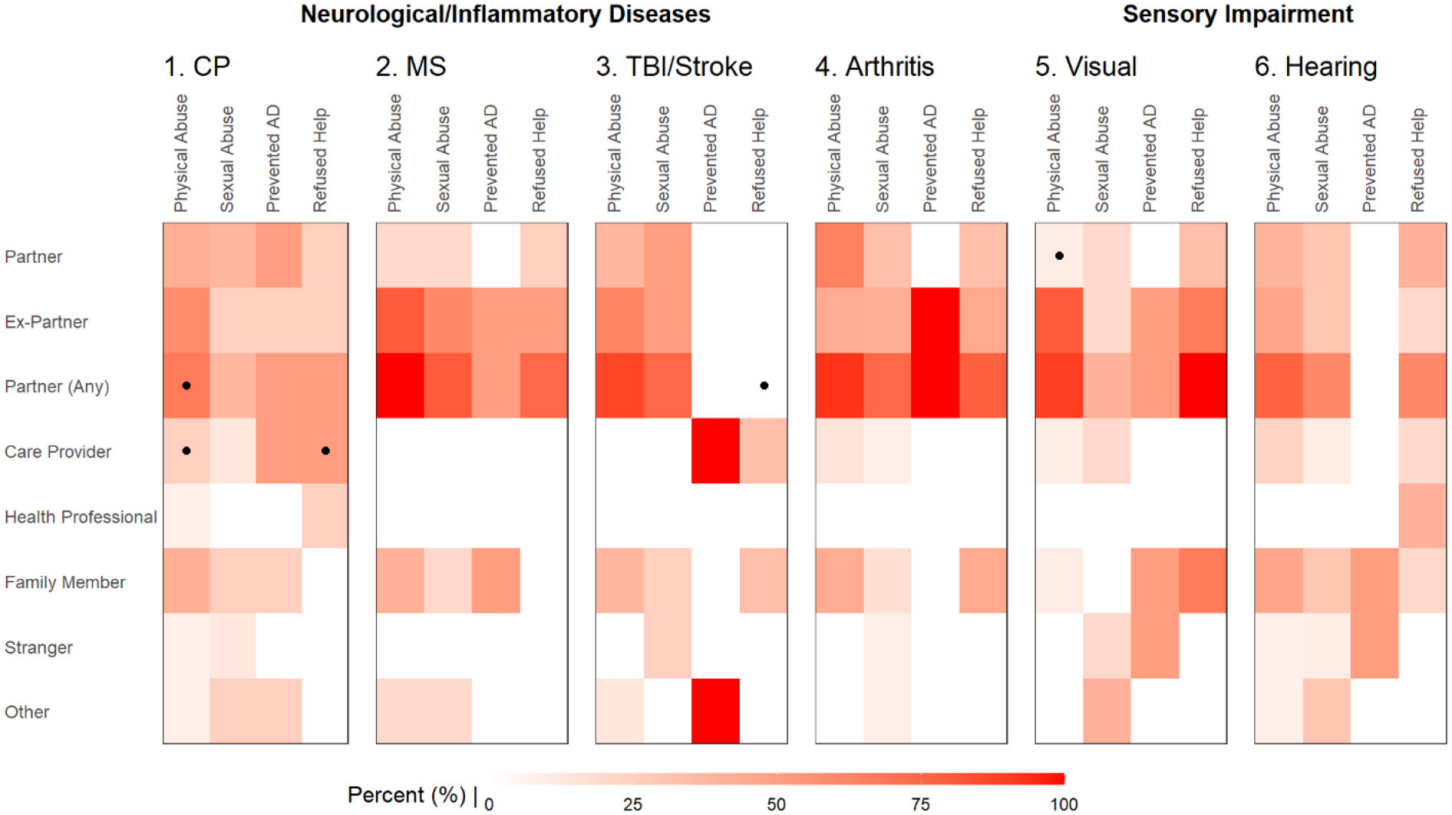
Comparing the frequency of relationships with perpetrators among women with different types of disabilities. Heatmap illustrating the reported frequency rate (%) of perpetrators among those experiencing physical abuse, sexual abuse, prevented use of assistive device (AD), and refused help of basic needs. Frequencies were compared between the groups using Fisher's exact test with *P* < 0.05 highlighted (dot). CP, cerebral palsy; MS, multiple sclerosis; TBI, traumatic brain injury.

Among those that responded to the follow-up questionnaire regarding their history of abuse (*n* = 57), most women reported physical/sexual abuse either from a partner (89.6%/62.9%) or family members (35.4%/17.1%) rather than strangers (4.2%/11.4%). As shown in Figure 3, women with CP had a higher risk of physical abuse and being denied assistance with basic needs from healthcare providers (*p* < 0.05). This is likely related to the frequent need for more essential personal assistance (e.g., dressing, toileting, and personal hygiene) compared with other disabilities (Figure 2). On the contrary, women with MS, arthritis, and visual impairment, were more likely to experience abuse by a partner (*p* = 0.02).

## Discussion

There is a considerable lack of data on the relationship between different types of violence and forms of disability (14). The investigation faces fundamental challenges related to the victims’ commonly experienced barriers to seeking help, which is pronounced in women with disabilities due to the emphasized perception of shame, guilt, and inferiority as well as functional limitations (11, 13, 15). Particularly women with severe and acute-onset physical disabilities are underrepresented (16, 17). In addition, cultural barriers and sociodemographic differences influence the perception of abuse and disabilities (4, 15, 17), which also affects the reporting of mistreatment. Hence, screening strategies customized to common high-risk disability groups may improve the detection of ongoing victimization and ease access to support (2, 9, 12, 18, 19).

This study investigates the pattern of abuse among Caucasian and African- American women with disabilities living in the USA. Several confounding risk factors include living arrangements, education, poverty, and perpetuator-related characteristics, particularly substance abuse (5, 8). Due to comprehensive efforts to disseminate information regarding the study's objectives, convenience sampling might have led to potential selection bias during recruitment, although this likely does not affect the comparisons of frequencies between disability groups. Notably, being a multi-ethnic society, factors such as age, type of disability, ethnicity, and socioeconomic status intersect in complex ways to shape individuals' experiences of inequality and vulnerability to violence in the USA. While intersectionality is a critical framework for understanding these dynamics, the scope of our current analysis was shaped by the limitations of our sample. Due to the lack of statistical power, individuals with less common disabilities were excluded. Similarly, individuals not of Caucasian or African American ethnicities were also excluded from this analysis due to the small sample size, which limits both interpretability and heterogeneity. Therefore, our analytical approach focused on the subgroups with sufficient representation to allow for meaningful statistical analysis. We recognize this as a limitation and recommend that future research be designed with larger, more diverse samples to support intersectional analyses that more fully capture the complexity of these issues. Nevertheless, although limitations in study power did not allow for assessment of detailed interactions between subtypes and severities of abuse and other demographic and lifestyle factors, our analyses indicate that the nature of disability shapes the type of abuse.

It has been reported that the timing of disability onset and its progression can affect an individual's reliance on and susceptibility to abuse from certain perpetrators (e.g., care providers, intimate partners, and family members) (20). Moreover, chronic diseases may affect the risk and duration of abuse through psychological and social factors related to the management of the disease activity/progression (11). Accordingly, we could observe that women with CP were more often physically abused and refused assistance from care providers, likely due to their frequent interactions with the personnel, and a higher degree of personal care required (20). However, this was not the case for less debilitating conditions, where violence and neglect were predominantly perpetrated by intimate partners (2, 5, 11, 12).

According to our analyses, women with hearing impairment did not differ in type of abuse relative to other conditions. Nevertheless, they had the highest overall frequency of abuse, including more ongoing incidents at the time of response. Since deafness is frequently associated with muteness and impaired articulation, those with hearing impairment may be more susceptible to violence and remain in abusive relationships due to difficulties communicating and seeking help (21). Notably, women with hearing problems were generally less likely to need personal assistance, which is commonly associated with a higher risk for interpersonal violence. Instead, they were more likely to require assistance with their medication. Need for this kind of assistance may be attributed to an altered perception of time and/or oblivion associated with the reduction of external stimuli, or even a cognitive impairment. Indeed, it has been shown that hearing impairment is related to the risk for cognitive decline, brain atrophy and tau accumulation (22), and considered one of the greatest risks for dementia (23). Hence, based on our findings, it is tempting to speculate that cognitive impairment may not only enhance, but pose a higher risk for abuse than sensory or physical disability itself, thus calling for further research.

The current knowledge in the field is predominantly based on older studies indicating that the acts of violence/abuse are dependent on the type and severity of functional impairment and its required assistive services (11, 20). Similarly, a recent study from Massachusetts based on prospectively and retrospectively collected data, shows that over a third of women with MS (in both cohorts, respectively) had a history of abuse, while 15%–17% of them have experienced violence also during the previous year (24). In addition, women with MS were reportedly most likely abused by a partner (24). Of note, all these findings are consistent with our data regarding the same diagnostic group. Further, a recent meta-analysis on sexual violence against persons with disabilities that included 68 studies, and 12,427 participants revealed that the highest risk of being sexually victimized was associated with sensory disability (25). This is also consistent with our report of the highest rates of physical or sexual abuse in women with hearing impairment. Thus, given the fact that our study is based on the data collected nearly two decades earlier, it seems that the prevalence of abuse in these disability groups remains consistent over time, despite public awareness and societal efforts.

## Conclusion

The lack of data on mistreatment of women with disabilities may implicate higher risks for abuse in this vulnerable population. Further research focusing on patterns of abuse specific to certain disability subtypes (11, 16, 19) is likely to enable more targeted interventions (14, 16). Lastly, future studies should identify the long-term psychological, physical, and social implications of abuse (7, 9), and how these relate to standard clinical care of the underlying medical conditions.

## Data Availability

Publicly available datasets were analyzed in this study. This data can be found here: https://doi.org/10.3886/ICPSR03414.v1.

## References

[1] World Health Organization. Violence Against Women. Geneva: World Health Organization (2024). https://www.who.int/news-room/fact-sheets/detail/violence-against-women (Accessed July 09, 2024).

[2] Plummer SB Findley PA. Women with disabilities’ experience with physical and sexual abuse: review of the literature and implications for the field. Trauma Violence Abuse. (2012) 13(1):15–29. 10.1177/1524838011426014 22070987

[3] Martin SL Ray N Sotres-Alvarez D Kupper LL Moracco KE Dickens PA Physical and sexual assault of women with disabilities. Violence Against Women. (2006) 12(9):823–37. 10.1177/1077801206292672 16905675

[4] Jewkes R. Intimate partner violence: causes and prevention. Lancet. (2002) 359(9315):1423–9. 10.1016/S0140-6736(02)08357-5 11978358

[5] Kyriacou DN Anglin D Taliaferro E Stone S Tubb T Linden JA Risk factors for injury to women from domestic violence. N Engl J Med. (1999) 341(25):1892–8. 10.1056/NEJM199912163412505 10601509

[6] Rodriguez E Lasch KE Chandra P Lee J. Family violence, employment status, welfare benefits, and alcohol drinking in the United States: what is the relation. J Epidemiol Community Health. (2001) 55(3):172–8. 10.1136/jech.55.3.172 11160171 PMC1731852

[7] Tolman RM Rosen D. Domestic violence in the lives of women receiving welfare. Violence Against Women. (2001) 7(2):141–58. 10.1177/107780120100700200

[8] Brownridge DA. Partner violence against women with disabilities: prevalence, risk, and explanations. Violence Against Women. (2006) 12(9):805–22. 10.1177/1077801206292681 16905674

[9] Campbell J Jones AS Dienemann J Kub J Schollenberger J O'Campo P Intimate partner violence and physical health consequences. Arch Intern Med. (2002) 162(10):1157–63. 10.1001/archinte.162.10.1157 12020187

[10] Eberhard-Gran M Schei B Eskild A. Somatic symptoms and diseases are more common in women exposed to violence. J Gen Intern Med. (2007) 22(12):1668–73. 10.1007/s11606-007-0389-8 17922169 PMC2219828

[11] Nosek MA Howland C Rintala DH Young ME Chanpong GF. National study of women with physical disabilities: final report. Sex Disabil. (2001) 19(1):5–40. 10.1023/A:1010716820677

[12] Oram S Khalifeh H Howard LM. Violence against women and mental health. Lancet Psychiatry. (2017) 4(2):159–70. 10.1016/S2215-0366(16)30261-9 27856393

[13] Milberger S Israel N LeRoy B Martin A Potter L Patchak-Schuster P. Violence against women with physical disabilities. Violence Vict. (2003) 18(5):581–91. 10.1891/088667003780928080 14695023

[14] Meyer SR Stöckl H Vorfeld C Kamenov K García-Moreno C. A scoping review of measurement of violence against women and disability. PLoS One. (2022) 17(1):e0263020. 10.1371/journal.pone.0263020 35100320 PMC8803172

[15] Hassouneh-Phillips D McNeff E. “I thought I was less worthy”: low sexual and body esteem and increased vulnerability to intimate partner abuse in women with physical disabilities. Sex Disabil. (2005) 23(4):227–40. 10.1007/s11195-005-8930-3

[16] Gilson SF DePoy E Cramer EP. Linking the assessment of self-reported functional capacity with abuse experiences of women with disabilities. Violence Against Women. (2001) 7(4):418–31. 10.1177/10778010122182532

[17] Nosek MA Hughes RB Taylor HB Taylor P. Disability, psychosocial, and demographic characteristics of abused women with physical disabilities. Violence Against Women. (2006) 12(9):838–50. 10.1177/1077801206292671 16905676

[18] Chang JC Martin SL Moracco KE Dulli L Scandlin D Loucks-Sorrel MB Helping women with disabilities and domestic violence: strategies, limitations, and challenges of domestic violence programs and services. J Womens Health (Larchmt). (2003) 12(7):699–708. 10.1089/154099903322404348 14583110

[19] McFarlane J Hughes RB Nosek MA Groff JY Swedlend N Dolan Mullen P. Abuse assessment screen-disability (AAS-D): measuring frequency, type, and perpetrator of abuse toward women with physical disabilities. J Womens Health Gend Based Med. (2001) 10(9):861–6. 10.1089/152460901753285750 11747680

[20] Saxton M Curry MA Powers LE Maley S Eckels K Gross J. Bring my scooter so I can leave you. Violence Against Women. (2001) 7(4):393–417. 10.1177/10778010122182523

[21] Anderson ML Leigh IW Samar VJ. Intimate partner violence against deaf women: a review. Aggress Violent Behav. (2011) 16(3):200–6. 10.1016/j.avb.2011.02.006

[22] Wang HF Zhang W Rolls ET; Alzheimer's Disease Neuroimaging Initiative, Li Y Wang L Hearing impairment is associated with cognitive decline, brain atrophy and tau pathology. eBioMedicine. (2023) 8:e329–38. 10.1016/S2468-2667(23)00048-8 PMC9649369 36356475

[23] Jiang F Mishra SR Shrestha N Ozaki A Viranin SS Bright T Association between hearing aid use and all-cause and cause-specific dementia: an analysis of the UK biobank cohort. Lancet Public Health. (2023) 8:e329–38. Published Online April 13, 2023. 10.1016/S2468-2667(23)00048-8 37062296

[24] Pol-Patil J Glanz B Safar L Misasi E Manieri MC Shanahan R MeTooMS: sexual, physical, and emotional abuse experience among women with multiple sclerosis. Multiple Sclerosis Journal. (2023) 29(2):287–94. 10.1177/13524585221122169 36154526

[25] Mailhot Amborski A Bussières EL Vaillancourt-Morel MP Joyal CC. Sexual violence against persons with disabilities: a meta-analysis. Trauma Violence Abuse. (2022) 23(4):1330–43. 10.1177/1524838021995975 33657931 PMC9425723

